# miR-18a counteracts AKT and ERK activation to inhibit the proliferation of pancreatic progenitor cells

**DOI:** 10.1038/srep45002

**Published:** 2017-03-23

**Authors:** Xuyan Li, Zhenwu Zhang, Yunchao Li, Yicheng Zhao, Wenjun Zhai, Lin Yang, Delin Kong, Chunyan Wu, Zhenbao Chen, Chun-Bo Teng

**Affiliations:** 1 College of Life Science, Northeast Forestry University, Harbin, 150040, China; 2 College of Life Sciences and Agriculture and Forestry, Qiqihar University, Qiqihar, 161006, China; 3 Faculty of Health Sciences, University of Macau, Taipa, Macau, People’s Republic of China

## Abstract

Activation of endogenous stem/progenitor cells to repair injured tissues is an ideal option for disease treatment. However, adult pancreatic progenitor cells remain in a quiescent state *in vivo*. Thus, it is difficult to stimulate proliferation and differentiation in these progenitor cells, and the cause remains elusive. miR-17-92 cluster miRNAs are highly conserved in mammals and are expressed in multiple tissue stem/progenitor cells, but their role in pancreatic progenitor cells are less well known. In the present study, we demonstrate that miR-18a, but not the other members of the miR-17-92 gene cluster, inhibits the proliferation of pancreatic progenitor cells *in vitro* and *ex vivo*. miR-18a inhibits proliferation of adult pancreatic progenitor cells through arresting the cell cycle at G1 stage, indicating that miR-18a plays a role in keeping the adult pancreatic progenitor cells in quiescence. miR-18a inhibits pancreatic progenitor proliferation by targeting the gene expressions of connective tissue growth factor (CTGF), neural precursor cell expressed, developmentally down-regulated 9 (Nedd9), and cyclin dependent kinase 19 (CDK19), as well as by suppressing activation of the proliferation-related signaling pathways phosphatidylinositol 3-kinase–protein kinase B (PI3K/AKT) and extracellular signal-regulated kinase (ERK).

Stem/progenitor cells observed in multiple tissues of mammals exhibit self-renewal and multipotent capacity, which are responsible for organ growth and tissue maintenance. Based on their excellent proliferative and differentiation capabilities, tissue stem/progenitor cells provide an alternative approach for regeneration medicine through grafting exogenous stem cells disposed *in vitro* or activating endogenous stem/progenitor cells *in vivo*^1^,^2^. The strategy of endogenous stem/progenitor cell motivation is safer and leads to fewer immuno-rejection problems. It has been proposed as an ideal option for regenerative treatment against multiple diseases^3^,^4^,^5^.

The pancreas, an organ with both endocrine and exocrine portions, plays a pivotal role in vertebrate nutrient metabolism and glucose balance regulation. Autoimmune destruction of beta-cells causes type 1 diabetes, and insulin resistance and insulin deficiency lead to type 2 diabetes. It has been demonstrated that progenitor cells exist in the adult pancreas, and these cells could be activated to proliferate and differentiate into beta cells under the condition of pancreatic duct ligation or culture *in vitro*^6^,^7^,^8^,^9^. However, in normal or islet-specific injury animals, it was difficult to activate the proliferation and differentiation of pancreatic progenitor cells into beta cells^10^,^11^.

During embryogenesis, pancreatic progenitor cell proliferation is stimulated by mesenchyme-secreted mitogenic factors, such as fibroblast growth factors (FGFs) and Wingless and INT-1 (Wnts), through activation of extracellular signal-regulated kinase (ERK), serine/threonine-specific protein kinase B (PKB/AKT), or beta-catenin signaling, respectively^12^,^13^. In contrast, pancreatic progenitor cell proliferation is arrested by transforming growth factor beta (TGF-β) signal through promoting the expression of cyclin-dependent kinase inhibitor P57 and is repressed through the expression of endocrine master transcription factor Ngn3, which subsequently promotes endocrine cell differentiation^14^,^15^,^16^. Nevertheless, the mechanisms underlying inhibition of proliferation of adult pancreatic progenitor cells are not well known.

microRNAs (miRNAs) have been implicated in the regulation of organ development and cell proliferation^17^. miR-17-92 gene cluster, including 7 miRNAs, miR-18a, miR-92-1, miR-19b-1, miR-20a, miR-19a, miR-17-3p, and miR-17-5p, has been demonstrated to be onco-miR in multiple tumors, and it also plays an important role in organ development^18^,^19^. miR-17-92 deficient mice died shortly after birth, and death was accompanied by lung hypoplasia, ventricular septal defects, and a greatly reduced number of B cells^20^. Conditional knockout of miR-17-92 in the developing brain’s cortex restricts neural stem cell proliferation, suppresses primary cortical neural progenitor expansion, and promotes their transition to intermediate progenitors^21^. Further researches showed that miR-17, miR-19, miR-20, and miR-92 regulate the self-renewal and differentiation of neural stem/progenitor cells^22^,^23^,^24^.

In previous studies, we have reported that miR-17-92 cluster member miR-18a and miR-19b are expressed in embryonic buds and adult pancreatic progenitor cells. miR-18a could target pancreas specific transcription factor1a (Ptf1a) expression^25^, and miR-19b could target NeuroD expression, thus regulating the differentiation and function of pancreatic beta-cells^26^. A single miRNA is capable of targeting expression of multiple genes, and its function is multifaceted. In this paper, we disclose the role of miR-17-92 miRNA members in controlling proliferation of adult pancreatic progenitor cells and explore the mechanism of miR-18a underlying the regulation of proliferation of pancreatic progenitor cells.

## Results

### Effect of miR-17-92 cluster miRNAs on the proliferation of pancreatic progenitor cells

**Figure 1:**
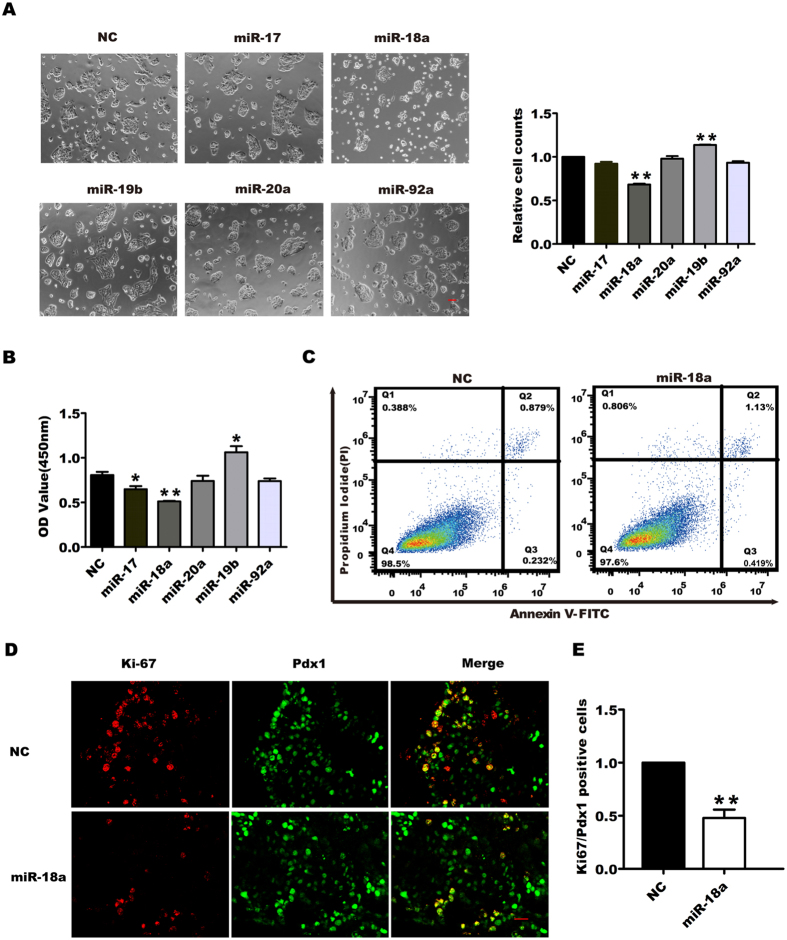
Effect of miR-17-92 cluster miRNAs on the proliferation of pancreatic progenitor cells. Pancreatic progenitor cells were transfected with the negative control miRNA (NC), miR-17, miR-18a, miR-20a, miR-19b, or miR-92a (50 nM). Seventy-two hours later, the transfected cells were photographed by a microscope with a CCD camera (Scale Bar 100 μm) and clounted using an auto cell counter (**A**), or detected by CCK8 assay (**B**). For the cells transfected with miR-18a or NC, after transfection for 48 h, they were fixed and double-stained by Annexin V-FITC and propidium iodide (PI) to detect cell apoptosis by flow cytometry (**C**). The E13.5 dorsal pancreatic buds were dissected and cultured for 24 h and then were transfected with NC or miR-18a (1 μM, respectively) overnight. Seventy-two hours later, the explants were fixed and embedded to be frozen-sectioned consecutively. The sections were double immuno-stained using antibodies against Pdx1 and Ki-67, and images were taken using a laser confocal microscope (Scale Bar 20 μm) (**D**). The proliferative ratios were determined by double positive cells divided by Pdx1-positive cells. The data from the miRNA transfected groups were normalized to the control group (E). *P < 0.05, **P < 0.01.

To determine the effects of miR-17-92 on the proliferation of pancreatic progenitor cells, we separately transfected mimics of miR-17, miR-18a, miR-19b, miR-20a, or miR-92a to the cultured pancreatic progenitors (Supplementary Fig. S1), which we isolated and detected as reported previously^[24]^. Cell counting results showed that, when the cells were transfected at a low density (30-40% confluence), the cell number significantly decreased in miR-18a transfection wells, but increased in miR-19b transfection wells, as compared with the negative control. However, the other miR-17-92 members exerted little effect on the cell number of pancreatic progenitors (Fig. 1A). The CCK8 assay confirmed that miR-18a suppressed, whereas miR-19b increased, pancreatic progenitor cell numbers compared to the negative control or the other 17-92 cluster miRNAs (Fig. 1B). To determine the mechanism underlying the decrease in cell numbers produced by miR-18a overexpression, we performed cell apoptosis analysis by flow cytometry. As shown in Fig. 1C, miR-18a overexpression did not significantly promote apoptosis in pancreatic progenitor cells, indicating that miR-18a decreased cell number through inhibiting pancreatic progenitor cell proliferation.

To confirm that miR-18a inhibited proliferation of pancreatic progenitor cells, we isolated the E13.5 embryonic pancreatic explants and transfected them with miR-18a or negative control (NC) for 72 h (Fig. 1D). The results of co-staining using antibodies against pancreatic duodenal homeobox 1 (Pdx1) and Ki-67 showed that miR-18a overexpression significantly decreased Ki67-positive cells in the Pdx1-expressing cell population, relative to the negative control (*P* < 0.01), suggesting that miR-18a could inhibit the proliferation of pancreatic progenitor cells *ex vivo*.

### miR-18a inhibits the proliferation of pancreatic progenitor cells through blocking cell cycle progression

**Figure 2:**
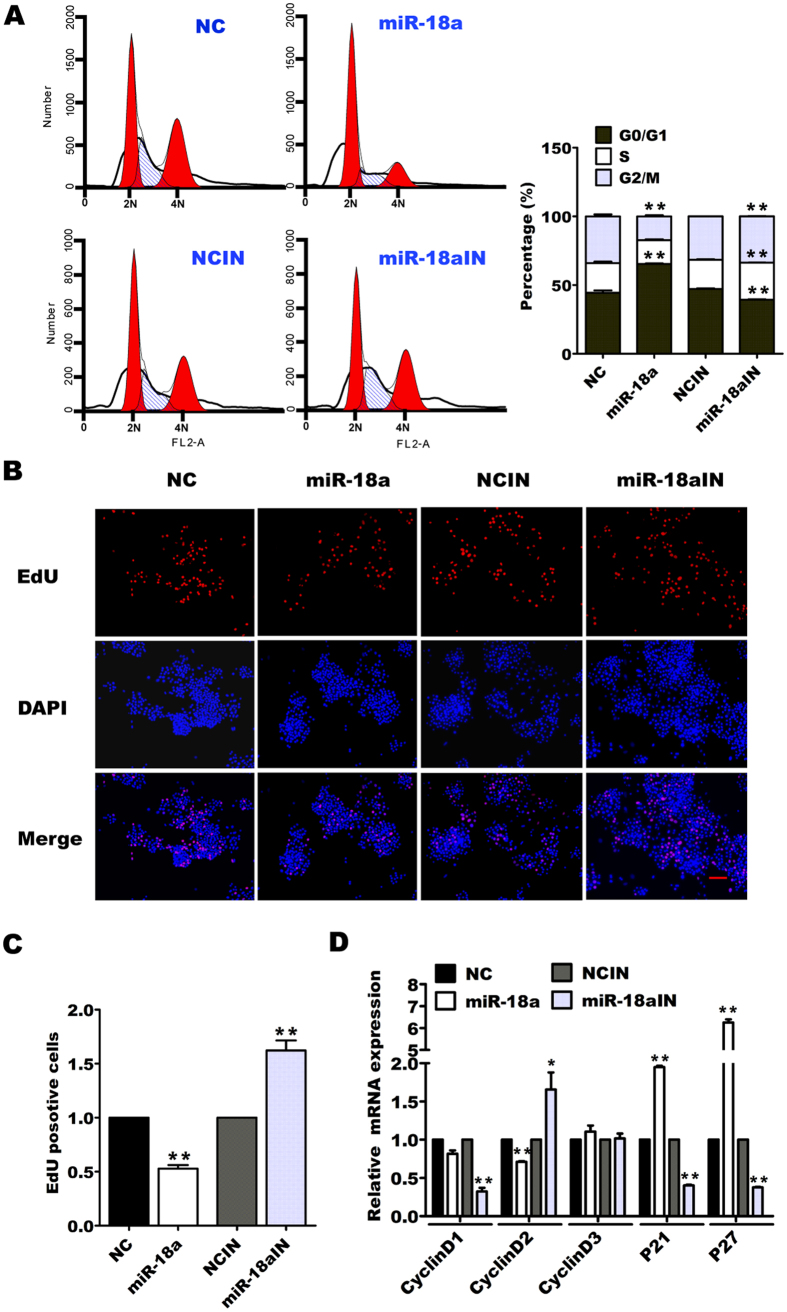
Effect of miR-18a on the cell cycle progression of pancreatic progenitor cells. Pancreatic progenitor cells were transfected with NC, miR-18a (50 nM), miR-18aIN, or NCIN (100 nM). Fourty-eight hours later, the cell cycles were analyzed using flow cytometry (**A**). Seventy-two hours later, the transfected cells were incubated in the culture medium with EdU (10 μM) for 4 h, and then detected using an EdU labeling/detection kit (Scale Bar 100 μm) (**B**), The EdU-positive cells were divided by the DAPI-positive cells (**C**). For the gene expression assay, after transfection for fourty-eight hours later, the cellular total RNAs were extracted and reverse transcripted to perform qRT-PCR (**D**). The data from the miR-18a transfected groups were normalized to that of control group. *P < 0.05, **P < 0.01.

Our data showed that, among miR-17-92 cluster members, only miR-18a inhibited the pancreatic progenitor cell proliferation. We introduced a miR-18a inhibitor (miR-18aIN) to the pancreatic progenitor cells and found that the miR-18a inhibitor neutralized endogenous miR-18a and reversed the inhibition of proliferation (Supplementary Fig. S2). To further explore the cause of cell proliferation inhibition by miR-18a, we analyzed the effect of miR-18a or miR-18a inhibitor on the cell cycle of cultured pancreatic progenitor cells. FACs analysis showed that miR-18a overexpression caused the majority of pancreatic progenitor cells to remain at the G1 stage, whereas miR-18a inhibitor decreased G1 stage, and increased S and G2 stage cell numbers, compared with the inhibitor control (NCIN) (Fig. 2A). 5-Ethynyl-2′- deoxyuridine (EdU) incorporation assay revealed that miR-18a decreased EdU incorporation into the pancreatic progenitor cells, but the miR-18a inhibitor increased EdU uptake (Fig. 2B,C). Gene expression analysis showed that miR-18a downregulated the mRNA expression of cell cycle genes, including *cyclinD1* and *2,* and significantly upregulated that of cyclin-dependent kinase inhibitors *P21* and *P27* (Fig. 2D). In contrast, miR-18a inhibitor increased the expression of *cyclinD2* and decreased the expression of *P21* and *P27* after transfection with miR-18a inhibitor for 48 h (Fig. 2D). These results demonstrate that miR-18a inhibits the proliferation of pancreatic progenitor cells by blocking cell cycle progression.

### miR-18a targets multiple proliferation-related genes in pancreatic progenitor cells

**Figure 3:**
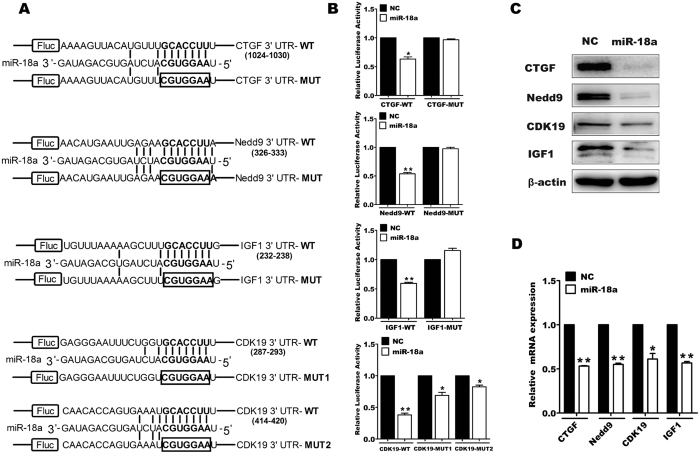
Analysis of the target genes of miR-18a in pancreatic progenitor cells. Schematic of the base pairing of miR-18a and its predicted target sequences on the 3′UTR of CTGF, Nedd9, IGF1, and CDK19 (**A**). miR-18a or NC, pMIR-target 3′UTR-luciferase or pMIR-target 3′UTR mut-luciferase, and Renilla pRL-SV40 vectors were co-transfected into 3T3 cells. Forty-eight hours later, Luciferase activity was determined using a Dual-Luciferase Reporter Assay System Kit. All Firefly luciferase values were normalized to the co-transfected Renilla luciferase values (**B**). Pancreatic progenitor cells were transfected with miR-18a or NC. Seventy-two hours after transfection, western blots were performed to determine the expression of CTGF, Nedd9, CDK19, and IGF1. β-actin was used as an endogenous control (**C**). Pancreatic progenitor cells were transfected with miR-18a or NC. Forty-eight hours after transfection, real-time quantitative RT-PCR was performed to determine the expression of *CTGF, Nedd9, CDK19*, and *IGF1* mRNA (**D**). The data from the miRNA transfected groups were normalized to that of control group. *P < 0.05, **P < 0.01. Uncropped images for the blots were shown in Supplementary Figure S4.

To elucidate the mechanism by which miR-18a repressed the proliferation of pancreatic progenitor cells, we predicted the candidate target genes of miR-18a via TargetScan algorithm (TargetScan 6.0, http://wwww.targetscan.org) and focused on four potential target genes including connective tissue growth factor (CTGF), neural precursor cell expressed, developmentally down-regulated 9 (Nedd9), cyclin dependent kinase 19 (CDK19), and insulin like growth factor 1 (IGF1) (Fig. 3A). CTGF is a secreted signaling molecule, usually crosstalking with the TGF-β signaling pathway, and is reported to regulate the proliferation of pancreatic cancer cells^27^. Nedd9 is an intracellular scaffolding protein that serves as a downstream gene of the Wnt/β-catenin signaling pathway^28^, CDK19, a homologous protein of CDK8 (both proteins are components of the mediator complex), uploads the RNA polymerase II transcription machinery and gene-specific transcription factors^29^. It has been reported that CTGF^30^, Nedd9^31^, and CDK19^31^ genes are targets of miR-18a in human cancer cells, and IGF1^32^ is a miR-18a target in deer antler. Whether CTGF, Nedd9, CDK19, and IGF1 are miR-18a targets in mouse pancreatic progenitor cells is still unknown.

We co-transfected the pMIR-report vectors containing the wild-type or mutated 3′UTR sites of CTGF, Nedd9, IGF1, and CDK19 with miR-18a or the negative control mimics, into 3T3 cells. As shown in Fig. 3B, the luciferase activities with the wild-type 3′UTR sequences of CTGF, Nedd9, IGF1, and CDK19 were all dramatically reduced by miR-18a introduction, but when the targeting sites of the four genes were mutated, the luciferase activities were nearly rescued. In order to detect the effects of miR-18a on the gene expression of the four candidate target genes, we performed qRT-PCR and western blot analysis on miR-18a transfected pancreatic progenitor cells. As shown in Fig. 3C and D, both protein and mRNA levels of CTGF, Nedd9, CDK19, and IGF1 were obviously inhibited. These results indicate that miR-18a could target the expressions of the four predicted target genes in pancreatic progenitor cells by inhibiting both transcription and translation.

### Knock-down of miR-18a target genes inhibits the proliferation of pancreatic progenitor cells

**Figure 4:**
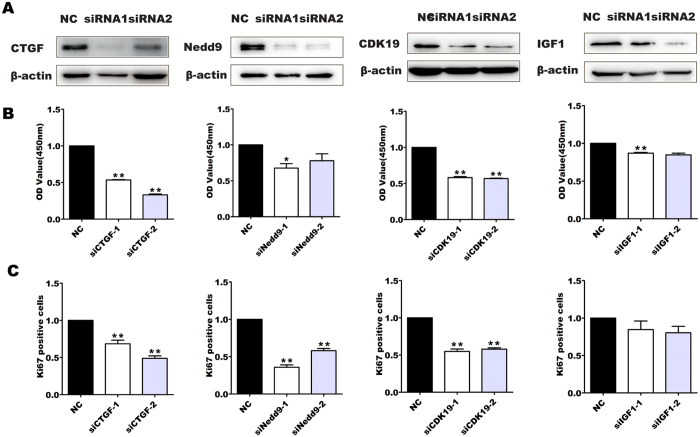
Effect of miR-18a target gene knock-down on the proliferation of pancreatic progenitor cells. Pancreatic progenitor cells were transfected with siCTGF-1/2, siNedd9-1/2, siCDK19-1/2, and siIGF1-1/2 or sicontrol (NC) for 72 h, and the knockdown of these genes was confirmed by western blot assay (**A**). The proliferation of pancreatic progenitor cells that knocked down the target genes were evaluated by CCK8 assay (**B**) and Ki67 immunofluorescent staining (**C**). The Ki67-positive cells were divided by DAPI-positive cells. The data from the siRNA transfected groups were normalized to that of control group. *P < 0.05, **P < 0.01. Uncropped images for the blots were shown in Supplementary Figure S5.

We have demonstrated that increased miR-18a repressed the proliferation of pancreatic progenitor cells and that miR-18a could target the expression of CTGF, Nedd9, CDK19, and IGF1 in pancreatic progenitor cells. However, whether the expression inhibition of CTGF, Nedd9, CDK19 or IGF1 is responsible for the proliferation suppression of miR-18a is unclear. To answer this question, we knocked down CTGF, Nedd9, CDK19, or IGF1 separately via transfection of siRNAs into the pancreatic progenitor cells for 72 h (Fig. 4A). Both CCK8 assays and Ki-67 immunostaining showed that knockdown of CTGF19, Nedd9, and CDK19, but not IGF1 significantly decreased the proliferation of pancreatic progenitor cells (Fig. 4B,C), indicating that CTGF, Nedd9, and CDK19, (but not IGF1) are the potential downstream effectors of miR-18a in repressing pancreatic progenitor proliferation.

### miR-18a suppresses activation of AKT and ERK signaling pathways in pancreatic progenitor cells

**Figure 5:**
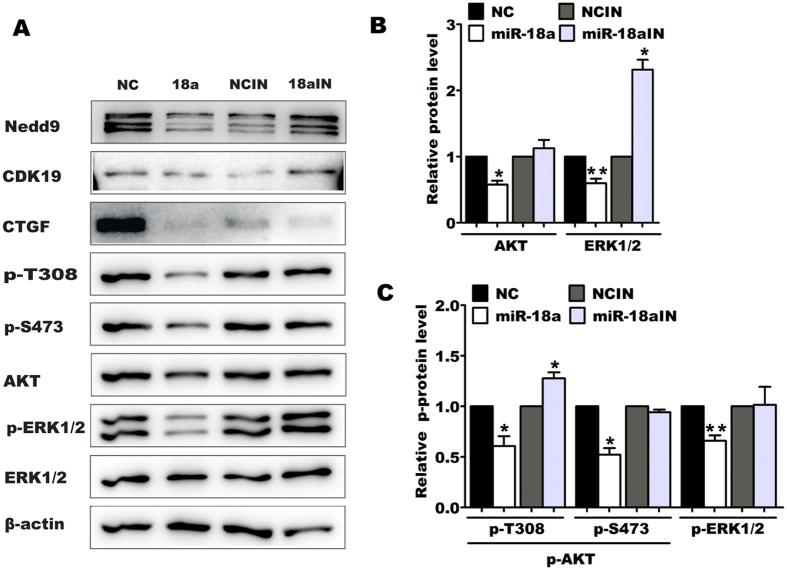
Effect of miR-18a on the phosphorylation of AKT and ERK1/2. Pancreatic progenitor cells were transfected with miR-18a or NC. Seventy-two hours later, the phosphorylation of AKT on threonine 308 (p-T308) and serine 473 (p-S473), and ERK1/2 (p-ERK1/2 at Thr202/Tyr204) were analyzed by western blot (**A**). β-actin was used as a loading control. The experiments were run in triplicate. The relative total protein (**B**) and phosphorylated protein levels (**C**) are represented as the mean ± SEM for the three independent experiments. *P < 0.05, **P < 0.01. Uncropped images for the blots were shown in Supplementary Figure S6.

Considering that the proliferation of embryonic and adult pancreatic progenitor cells depends on the involvement of growth factors and that activation of their downstream signaling pathways (PI3K/AKT and/or ERK pathways) are critical for cell proliferation regulation^33^,^34^, we proposed that the inhibition of proliferation and cell cycle progression in pancreatic progenitor cells produced by miR-18a might involve PI3K/AKT and/or ERK signaling pathways. As shown in Fig. 5A and C, miR-18a overexpression significantly repressed AKT phosphorylation at both threonine 308 (p-T308) and serine 473 (p-S473) and ERK1/2 phosphorylation at Thr202/ Tyr204 (p-ERK1/2) (Fig. 5A and C). Surprisingly, miR-18a also decreased the total protein level of AKT and ERK1/2 (Fig. 5A and B). However, when miR-18a inhibitor was transfected in pancreatic progenitor cells, both phosphorylated level of AKT and ERK1/2 were recovered or increased (Fig. 5A and C), and total protein level of AKT and ERK1/2, too (Fig. 5A and B). These results suggest that miR-18a inhibits pancreatic progenitor proliferation by blocking PI3K/AKT and ERK activity.

### Downregulation of CTGF, Nedd9 and CDK19 gene expression is responsible for miR-18a-induced PI3K/AKT and ERK inactivity and cell cycle arrest

**Figure 6:**
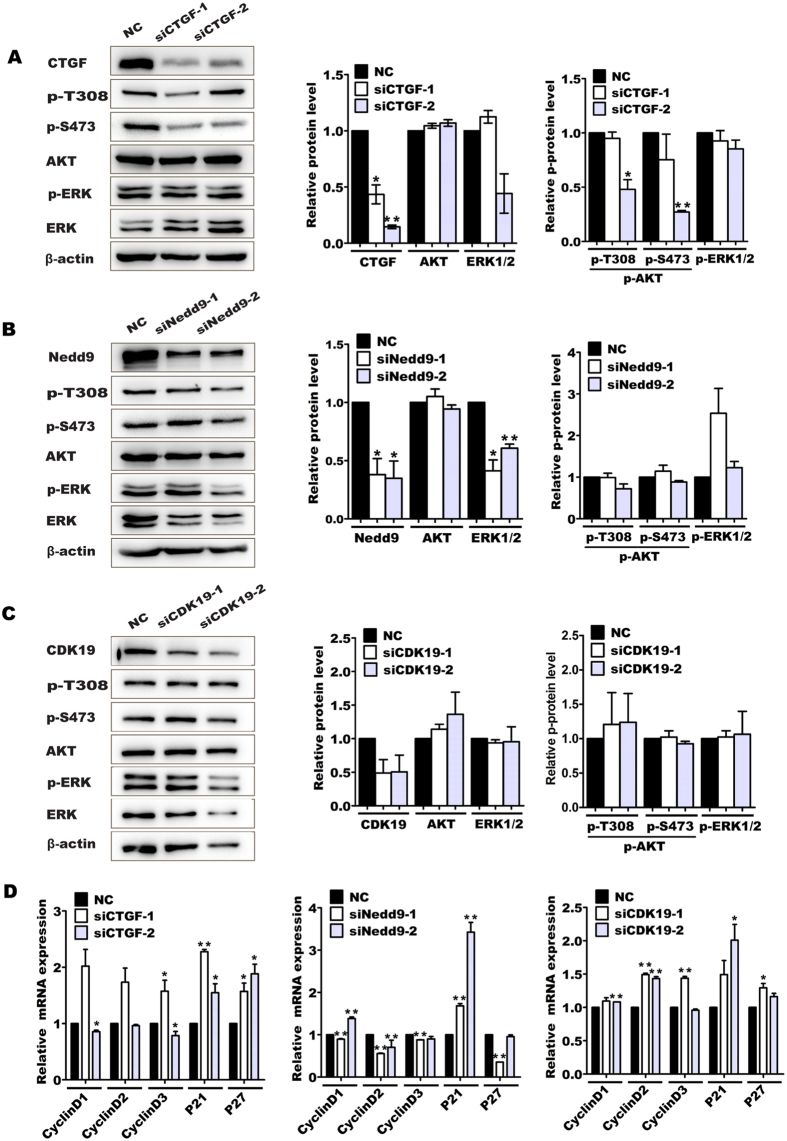
Effect of CTGF, Nedd9, or CDK19 knockdown on the phosphorylation of AKT and ERK1/2. Pancreatic progenitor cells were transfected with sicontrol (NC), siCTGF-1/2, siNedd9-1/2, and siCDK19-1/2. Seventy-two hours after transfection, the total and phosphorylated protein level of AKT (p-T308 and p-S473, respectively) and ERK1/2 (p-ERK1/2 at Thr202/Tyr204) were determined by western blot (**A–C**). β-actin was used as a loading control. The cell cycle gene expressions were detected by quantitative RT-PCR after gene knockdown for forty-eight hours (**D**). *P < 0.05, **P < 0.01. Uncropped images for the blots were shown in Supplementary Figure S7.

To determine which target genes of miR-18a are responsible for activation of PI3K/AKT and ERK signaling in pancreatic progenitor cells, we analyzed the phosphorylation level of AKT (p-T308 and p-S473) and ERK1/2 (p-ERK1/2 at Thr202/Tyr204) in pancreatic progenitor cells after CTGF, Nedd9, or CDK19 was knocked-down (Fig. 6). The results showed that CTGF knockdown significantly downregulated the phosphorylation level of AKT at both S473 and T308, and caused a little decrease in ERK1/2 phosphorylation (Fig. 6A). Nedd9 knockdown slightly decreased the level of phosphorylated AKT at S473 and T308, but exerted significantly effect on the total level of ERK1/2 (Fig. 6B). However, siCDK19s barely affected on the phosphorylations of AKT and of ERK1/2 (Fig. 6C). As anticipated, siIGF1s have little effect on the activity of ERK1/2 and AKT (Supplementary Fig. S3), indicating miR-18a inhibits PI3K/AKT and ERK activity mainly through targeting CTGF and Nedd9.

Because ERK1/2 and AKT are checkpoint kinases, inhibition of CTGF and Nedd9 gene expression may be responsible for the cell cycle arrest. To test this hypothesis, we further examined the expression of cell cycle genes after CTGF, Nedd9, or CDK19 knock-down in pancreatic progenitor cells (Fig. 6D). As anticipated, *P21* and/or *P27* mRNA expressions were increased after CTGF and Nedd9 were knocked-down. Athough siCDK19s did not decrease the expression of *cyclinDs* mRNA, they increased the expressions of *P21* and *P27* mRNA. Combined with the Ki-67 immuno-staining data, we infer that the decreases in CTGF, Nedd9 and CDK19 gene expression are responsible for miR-18a-mediated cell cycle arrest.

## Discussion

In the present study, we provide evidence that miR-18a, but not the other miR-17-92 gene cluster members, inhibits the proliferation of pancreatic progenitor cells and does not promote cell apoptosis. miR-18a inhibits the proliferation of pancreatic progenitor cells by targeting the gene expressions of CTGF, Nedd9, and CDK19, as well as by repressing the activation of PI3K/AKT and ERK. miR-18a suppresses the phosphorylation of AKT on S473 and T308 by targeting CTGF and Nedd9, further inhibiting cell cycle progression of pancreatic progenitor cells.

miR-18a is highly conserved in mammals and is expressed in multiple tissue stem/progenitor cells and cancers^18^,^19^. In several early studies, miR-18a had been regarded as an onco-miR because it was revealed to promote hepatoma carcinoma and nasopharyngeal carcinoma cell proliferation^35^,^36^. However, in recent reports, miR-18a was shown to inhibit the cell proliferation of bladder cancer, colorectal carcinoma, and gastric cancer^31^,^37^,^38^. Here, we revealed that miR-18a inhibits the proliferation of pancreatic progenitor cells *in vitro* and *ex vivo*, which is consistent with its role in mouse palatal mesenchymal cells^39^ and deer antler chondrocytes^32^, indicating that miR-18a acts as a proliferation suppressor in pancreas development.

Several studies have revealed that pancreatic progenitor cells remain in a quiescent state in adulthood^10^,^11^, whereas the cause for this is not well known. In this paper, we found that miR-18a inhibits proliferation of adult pancreatic progenitor cells by blocking cell cycle progression. In contrast, miR-18a inhibitor could facilitate pancreatic progenitor cells to enter into the S stage (Fig. 2A), indicating that miR-18a plays a role in keeping the adult pancreatic progenitor cells in quiescence. However, we did not observe a significant increase in cell number after miR-18a inhibitor was introduced into the cultured progenitor cells, suggesting that additional factors may be involved in preserving the quiescent state of adult pancreatic progenitor cells.

PI3K/AKT and/or ERK activation are essential for proliferation and cell cycle progression in diverse types of cells. In pancreatic progenitor cells, miR-18a inhibits the phosphorylation of AKT on both S473 and T308 sites and also influences the activity of ERK1/2 (phosphorylation at Thr202/Tyr204), indicating that miR-18a inhibits pancreatic progenitor proliferation and cell cycle progression by suppressing AKT and ERK activity. AKT activity leads to the downsteam phosphorylation of mammalian targets of rapamycin (mTOR), further regulating cyclinD1 and c-myc expression^40^. miR-18a inhibits AKT phosphorylation, thus reducing the sensitivity of mTORC1, and its two substrates S6K1 and 4E-BPl^38^,^41^, further repressing cell cycle progression by downregulating *cyclinD* gene expression and activating *P21* and *P27* (Fig. 2D).

Among the miR-18a target genes, CTGF was found to be expressed in the pancreatic epithelia on E12.5 and beta cells on E17.5 in mice. Using an inducible transgenic system to overexpress CTGF in mouse β cells during embryogenesis increased islet mass by promoting proliferation of immature β cells^42^. Knockdown of CTGF significantly decreased the proliferation of pancreatic progenitor cells, indicating an uncovered role of CTGF in pancreatic development. CTGF is capable of activating both ERK1/2 and AKT to promote myocardial hypertrophy^43^. However, in pancreatic progenitor cells, decrease of CTGF protein repressed the phosphorylated activity of AKT on S473 and T308, but exerted slightly influence on ERK1 activity. Although Nedd9 interference has been reported to decrease downstream activation of ras signaling, including ERK1/2^44^,^45^ and CDK19, which is found to be involved in extracellular signal-regulated kinase/mitogen-activated protein kinase (ERK/MAPK)^46^, knock-downs of Nedd9 and CDK19 exert a little effect on ERK phosphorylation in pancreatic progenitor cells. IGF1 interference also did not affect the AKT and ERK1/2 activity. Except for CTGF, Nedd9, CDK19, and IGF1, very few genes in ERK upstream pathway have been known as miR-18a targets, thus miR-18a decreased ERK phosphorylation may caused by its targeting the unidentified genes.

**Figure 7:**
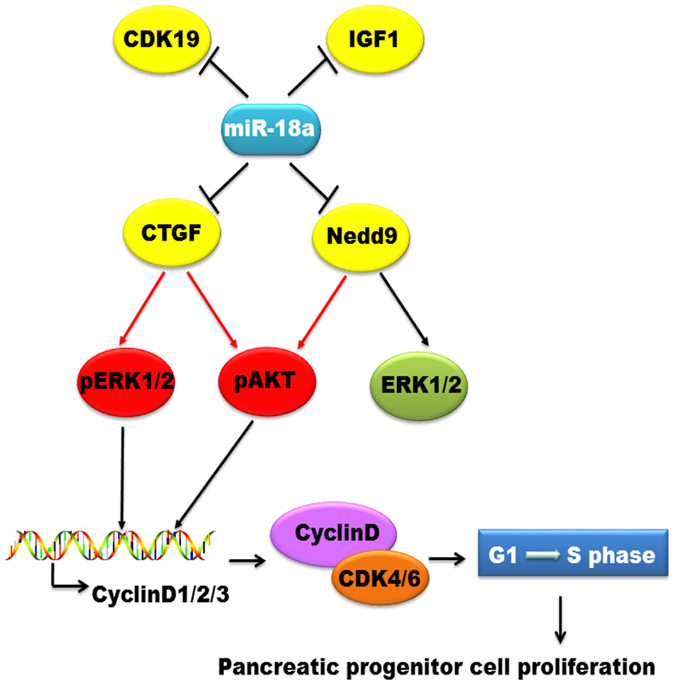
Diagram of the molecular mechanisms underlying miR-18a-induced inhibition of pancreatic progenitor cell proliferation. The red arrows represent activation by phosphorylation. The black arrows represent promotion whereas bar-headed lines represent inhibition.

In summary, we provide evidence that, in adult pancreatic progenitors, miR-18a inhibits cell proliferation and induces cell cycle arrest by targeting the expressions of CTGF, Nedd9, and CDK19, further counteracting the activation of AKT and ERK signaling (Fig. 7). miR-18a inhibitor reverses the target gene downregulation, promotes pancreatic progenitor cell entrance into the S stage, and partially activates the proliferation-related kinase AKT. This finding suggests that miRNA inhibitors may be used to relieve the endogenous progenitor cells of patients to cure their diabetes.

## Materials and Methods

### Animals

The protocol for use of animals was approved by the Animal Care and Ethics Committee of Northeast Forestry University, and all the procedures were carried out in accordance with the approved guidelines. Four- to eight-week-old ICR strain mice were purchased from Heilongjiang University of Chinese Medicine. Animals were housed with a 12 h light-dark cycle under specific-pathogen-free conditions and had free access to water and food pellets. The female mice were mated with fertile male mice. The morning of the discovery of the vaginal plug was designated as embryonic day (E) 0.5.

### Cell culture and transfection

The adult mouse pancreatic progenitor cells were isolated and cultured as described previously^26^. In brief, pancreatic progenitor cells were cultured in DMEM/F12 medium supplemented with 2% fetal bovine serum (FBS) (BI, Israel), 1% antibiotics (Sigma, St. Louis, MO, USA), 1% glutamine (Sigma, St. Louis, MO, USA), 1 × B_27_ (Invitrogen, California, USA), 20 ng/mL epithelial growth factor (EGF) (R&D, USA), 10 μg/mL Insulin (Sigma, St. Louis, MO, USA), and 50 μmol/L 2-mercaptoethanol (Sigma, St. Louis, MO, USA). The 5–7 passage pancreatic progenitor cells were transfected with miRNA negative control (NC), miR-17, miR-20a, miR-18a, miR-19b, miR-92a mimics (50 nM), miR-18a inhibitor (miR-18aIN) or miRNA inhibitor control (NCIN) (100 nM), and siRNAs or sicontrol (NC) (60 nM) using Lipofectamine RNAiMAX (Invitrogen, California, USA). miRNA overexpression or RNA interference efficiency was determined by quantitative RT-PCR 48 h after transfection, or by western blotting assay 72 h after transfection. The details of siRNAs and miRNA mimics/inhibitor are provided in Supplementary Table 1.

### Embryonic pancreatic bud microdissection and *in vitro* culture

At the 13.5th day of gestation, pregnant female mice were sacrificed to dissect each embryo under a stereomicroscope. The E13.5 dorsal pancreatic buds were isolated (denoted as pancreatic explants) and then digested in 1.5 mg/mL collagenase IV at 37 °C for 5 min to remove the mesenchyme. For growth, pancreatic explants were transferred to glass-bottomed 24 microwells coated with 10 μg/mL human fibronectin (hFN) and cultured in growth medium (DMEM/F12 medium supplemented with 10% fetal calf serum, 1% Pen/Strep and 1% Glutamine). The medium was changed every 2 days. The day of dissection was scored as day 0. E13.5 fetal pancreatic buds cultured for 24 h were transfected with miRNA control (NC) or miR-18a (1 μM) for 72 h using Lipofectamine RNAiMAX (Invitrogen, USA).

### CCK8 assay and cell counting

Cell number was assayed using cell counting kit-8 (CCK8) (Dojindo Laboratories, Japan). After the pancreatic progenitor cells were transfected for 72 h, 10 μL CCK8 reaction solution was added into cultured medium in each well for 2 h at 37 °C. The optical density was measured at 450 nm by a microplate reader (Sunrise™, Tecan, Männedorf, Switzerland). For cell counting assay, 72 h after transfection, all cells were trypsinized and counted using an automated cell counter (LUNA™ Automated Cell Counter, Logos Biosystems, Korea). Each experiment was repeated independently for three times.

### DNA incorporation assay

After miRNA transfection for 72 h, EdU was added to the culture media at a concentration of 10 μM for 4 h. The S stage cells were labeled by EdU and detected using an EdU labeling/detection kit (Ribobio Co., Ltd., Guangzhou, China) according to the manufacturer’s protocol. The experiment was repeated for three times.

### Cell cycle and apoptosis analysis by flow cytometry

After miRNA transfection for forty-eight hours, cellular apoptosis was analyzed by flow cytometry through Annexin V-FITC and propidium iodide (PI) double staining according to the protocol of the Apoptosis detection kit (Vazyme Biotech Co., Ltd., Nanjing, China). For cell cycle analysis, cells were harvested and fixed in 70% ethanol for 2 h at 4 °C. After washing with PBS, cells were treated with RNaseA (50 μg/mL) and stained with 50 mg/mL of propidium iodide (PI) for 30 min at 37 °C. Samples were analyzed using a flow cytometer (BD Accuri™ C6 Plus, BD Biosciences, New York, USA), and distribution of cell-cycle phases was analyzed using ModiFit LT 4.1 Software. All the experiments were conducted three times.

### Vector construction

The 3′UTR fragments of CTGF, Nedd9, IGF1, and CDK19 containing the putative miR-18a binding site were amplified by PCR. The corresponding mutant sequences of the miR-18a seed binding sites were obtained by overlapping PCR. The PCR products were cloned into the pMIR-report vector, and all constructs were confirmed by DNA sequencing.

### Dual luciferase reporter assay

The 3T3 cells were transfected with the pMIR-target 3′UTR-luciferase or pMIR-target 3′UTR mut-luciferase, Renilla pRL-SV40 control vector, miR-18a mimics, or miRNA negative control (NC) using Lipofectamine TM 2000 reagent (Invitrogen, California, USA). After 48 h of transfection, cells were lysed, and assays were performed using a Dual-Luciferase Reporter Assay System kit (Promega, Madison, WI, USA) on GloMaxTM 20/20 Luminometer (Promega, Sunnyvale, CA, USA). The data were analyzed by calculating the ratio of luminescence from the experimental firefly reporter to that from the control Renilla reporter. The ratios of miR-18a overexpression samples were normalized to the ratio of NC samples. All the assays were conducted three times.

### Quantitative real time PCR

The cellular total RNA was isolated using Tripure reagent (Roche, Indianapolis, USA) according to the manufacturer’s instructions. cDNA was synthesized with 1 μg total RNA using oligo (dT)_18_ (TransGen Biotech, Beijing, China). Faststart Universal SYBR Green Master (Roche, Indianapolis, USA) was used for real-time quantitative RT-PCR carried out in LightCycler 480 (LightCycler^®^ 480II Real-Time PCR System, Roche, Basel, Switzerland). Quantification was performed using the second derivative maximum method. Relative mRNA expression levels were normalized to the level of β-actin.

To detect miRNA expression, total RNAs were polyadenylated with ATP by Escherichia coli poly (A) polymerase (New England Biolabs, Inc., USA). The polyadenylated total RNAs were reverse transcribed with Reverse Transcription Reagents (TransGen Biotech, Beijing, China) and a poly (T) primer ligated with an adapter for miRNA quantitative assays. U6 was used as an internal control for miRNA detection. The sequences of all primers used are provided in Supplementary table 2. All the experiments were repeated independently for three times.

### Western blotting

Cellular total proteins were extracted with cell lysis buffer. Proteins were separated on 10% SDS-PAGE gels and electroblotted onto a nitrocellulose membrane. The membranes were blocked with 5% non-fat milk and incubated with primary antibodies. Antibodies against CTGF (cat. sc-14939, 1:500; Santa Cruz Biotechnology, TX, USA), Nedd9 (cat. #4044, 1:500; Cell Signaling Technology, MA, USA), CDK19 (cat. D161543, 1:1000; Sangon Biotech, Shanghai, China), IGF1 (cat. BA0939, 1:100, Boster, Wuhan, China), AKT (cat. #4691, 1:1000; Cell Signaling Technology, MA, USA), p-AKT 473 (cat. #4060, 1:1000; Cell Signaling Technology, MA, USA), p-AKT 308 (cat. #2965, 1:1000; Cell Signaling Technology, MA, USA), ERK1/2 (cat. #9102, 1:1000; Cell Signaling Technology, MA, USA), and p-ERK1/2 (cat. #9101, 1:1000; Cell Signaling Technology, MA, USA) were incubated overnight at 4 °C and HRP-conjugated secondary antibodies were incubated for 1 h at room temperature. The antigen-antibody complex was detected using Super ECL Detection Reagent (Tanon, Shanghai, China). Signal intensity was digitally quantified using Tanon-5200 (Tanon, Shanghai, China). The experiments were repeated at least three times.

### Immunofluorescent staining

Cells were fixed in 4% fresh paraformaldehyde for 30 min followed by permeabilization using 0.3% triton X-100. After blocking in 10% horse serum for 1 h, cells were incubated in the primary antibody rabbit anti-Ki67 (cat. NCL-Ki67P, 1:200, Novocastra Laboratories, Newcastle upon Tyne, UK) overnight at 4 °C. Incubation with secondary antibody was conducted for 1 h at room temperature. The cell nucleus was stained by DAPI (cat. C1002, 1:1000, Beyotime, Shanghai, China). Quantification of immunofluorescence staining was performed using Image-J software in a blinded manner.

The transfected pancreatic explants were fixed in 4% fresh paraformaldehyde for 30 min, embedded in OCT, and then frozen and cut into 10-μm-thick sections. Sections were subjected to immunofluorescent staining according to the protocol mentioned above. The first antibodies include rabbit anti-Ki67 and goat anti-Pdx1 (cat. ab47383, 1:10000; Abcam, Cambridge, UK). The pictures were captured using a laser confocal microscope (LSM 510 Meta, Zeiss, Jena, Germany). The experiments were run in triplicate.

### Statistical analysis

All the experiments were repeated independently three times. Statistical analyses were performed using Graphad Prism 5 software. Error bars represent mean ± SEM. Significance was determined with two-tailed, two-sample equal variance Student’s t-test. Differences less than 0.05 were considered significant and were indicated with an “*”. *indicates *P* < 0.05, **indicates *P* < 0.001.

## Additional Information

**How to cite this article**: Li, X. *et al*. miR-18a counteracts AKT and ERK activation to inhibit the proliferation of pancreatic progenitor cells. *Sci. Rep.* **7**, 45002; doi: 10.1038/srep45002 (2017).

**Publisher's note:** Springer Nature remains neutral with regard to jurisdictional claims in published maps and institutional affiliations.

## Supplementary Material

Supplementary Information
