# Supplementary material for: miR-18a counteracts AKT and ERK activation to inhibit the proliferation of pancreatic progenitor cells
Source: Sci Rep. 2017 Mar 23;7:45002. doi: 10.1038/srep45002 (PMC5362961; doi:10.1038/srep45002)
Supplement: Supplementary Information [file srep45002-s1.pdf]

Supplementary Information

miR-18a counteracts AKT and ERK activation to inhibit the proliferation of pancreatic progenitor cells

Xuyan Li[1, 2, #], Zhenwu Zhang[1, 3, #], Yunchao Li[1], Yicheng Zhao[1], Wenjun Zhai[1], Lin Yang[1], Delin Kong[1], Chunyan Wu[1], Zhenbao Chen[1] &Chun-Bo Teng[1, *]

[1] College of Life Science, Northeast Forestry University, Harbin, 150040, China

[2] College of Life Sciences and Agriculture and Forestry, Qiqihar University, Qiqihar, 161006, China

[3] Faculty of Health Sciences, University of Macau, Taipa, Macau, People's Republic of China

Supplementary Table 1.

miRNA mimics/inhibitor and siRNAs list used in this experiment.

**miRNAs/siRNAs**	**Sense (5´-3´)**	**Antisense (5´-3´)**
NC	UUCUCCGAACGUGUCACGUTT	ACGUGACACGUUCGGAGAATT
mmu-miR-18a	UAAGGUGCAUCUAGUGCAGAUAG	AUCUGCACUAGAUGCACCUUAUU
NCIN	CAGUACUUUUGUGUAGUACAA	
mmu-miR-18aIN	CUAUCUGCACUAGAUGCACCUUA	
mmu-miR-17	CAAAGUGCUUACAGUGCAGGUAG	ACCUGCACUGUAAGCACUUUGUU
mmu-miR-20a	UAAAGUGCUUAUAGUGCAGGUAG	ACCUGCACUAUAAGCACUUUAUU
mmu-miR-19b	UGUGCAAAUCCAUGCAAAACUGA	AGUUUUGCAUGGAUUUGCACAUU
mmu-miR-92a	UAUUGCACUUGUCCCGGCCUG	GGCCGGGACAAGUGCAAUAUU
siCTGF-1	GGUGAUAAAGCUAUGUAUUdtdt	AAUACAUAGCUUUAUCACCdtdg
siCTGF-2	CAAUACCUUCUGCAGACUGGAdtdt	UCCAGUCUGCAGAAGGUAUUGdtdt
siNedd9-1	CCUACCAGAAUCAGGGAAUUU	AAAUUCCCUGAUUCUGGUAGG
siNedd9-2	CCCACCAGAUUCUAAGCCAAA	UUUGGCUUAGAAUCUGGUGGG
siCDK19-1	AUGUAGCUAAGUCUACUUUAA	UUAAAGUAGACUUAGCUACAU
siCDK19-2	GCUUGUAGAGAGAUUGCACUU	AAGUGCAAUCUCUCUACAAGC
siIGF1-1	GCUGUGAUCUGAGGAGACUGGAGAU	AUCUCCAGUCUCCUCAGAUCACAGC
siIGF1-2	CAGCCUUCCAACUCAAUUAdTdT	UAAUUGAGUUGGAAGGCUGdTdT

Supplementary Table 2.

Primers used for gene expression detection in this experiment.

**Primer name**	**Sequences (5´-3´)**
CTGF Forward	GGGCCTCTTCTGCGATTTC
CTGF Reverse	ATCCAGGCAAGTGCATTGGTA
Nedd9 Forward	ATGTGGGCGAGGAATCTTATGG
Nedd9 Reverse	TTCCCTGGGACAATGCCTTG
CDK19 Forward	GGTCAAGCCTGACAGCAAAGT
CDK19 Reverse	TTCCTGGAAGTAAGGGTCCTG
IGF1 Forward	CATGTGCTGGCAGTATAACCC
IGF1 Reverse	TCGGGAGGCTTGTTCTCCT
U6 Forward	CGCTTCGGCAGCACATATAC
U6 Reverse	TTCACGAATTTGCGTGTCAT
Ambion Reverse	GCGAGCACAGAATTAATACGAC
miR-17-5p Forward	CAAAGTGCTTACAGTGCAGGT
miR-20a-5p Forward	CGTAAAGTGCTTATAGTGCAGG
miR-18a-5p Forward	CGTAAGGTGCATCTAGTGCAGAT
miR-19b-3p Forward	TGTGCAAATCCATGCAAAACT
miR-92a-3p Forward	TATTGCACTTGTCCCGGC
CyclinD1 Forward	TGGGACATAGCATCACAGCAG
CyclinD1 Reverse	CAAGACGGAACACTAGAACCTAACA
CyclinD2 Forward	TTCTGTGGTCTCGGGTGTATTTAG
CyclinD2 Reverse	GAGTGTGTCTGTCAAGTTCTGTGGG

CyclinD3 Forward	TTTTGAGTCCCTCTTCTGTCCG
CyclinD3 Reverse	AGTGTATGGGTTCTTGCTCTGGC
P21 Forward	GGCACCATGTCCAATCCTG
P21 Reverse	AAGTCAAAGTTCCACCGTTCTC
P27 Forward	CAGGCAAACTCTGAGGACCG
P27 Reverse	GGGGAACCGTCTGAAACATT

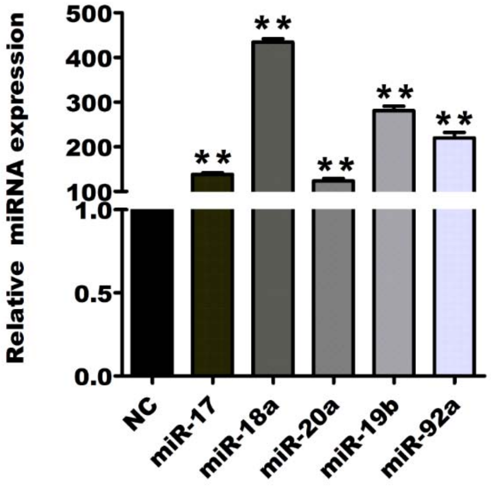

Supplementary Fig. S1. Overexpression of miR-17-92 cluster member in pancreatic progenitor cells. Pancreatic progenitor cells were transfected with the negative control miRNA (NC), miR-17, miR-18a, miR-20a, miR-19b, or miR-92a (50 nM). Forty-eight hours later, the level of miRNAs was detected by quantitative RT-PCR. The data from the miRNA transfected groups were normalized to that of control group.

A

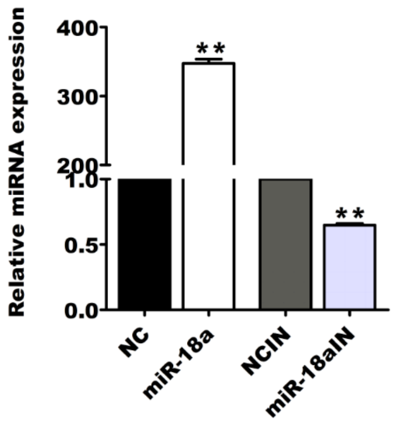

B

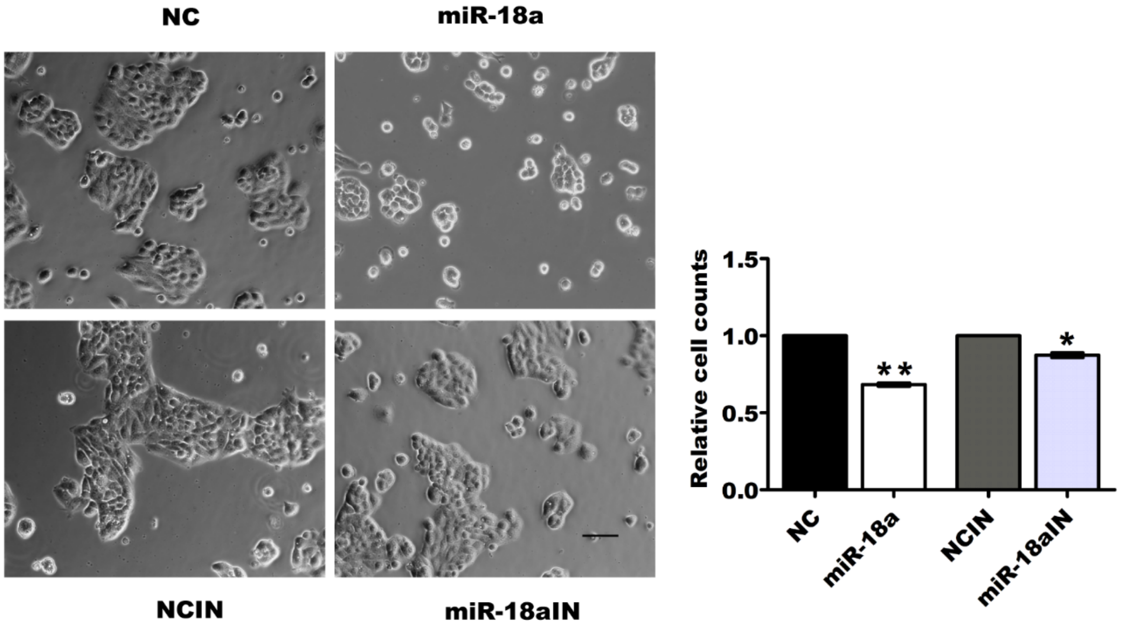

Supplementary Fig. S2. Effect of miR-18a and miR-18a inhibitor on the proliferation of pancreatic progenitor cells. Pancreatic progenitor cells were transfected with the control miRNA, miR-18a (50 nM, respectively), miR-18a inhibitor (miR-18IN), or the miRNA inhibitor control (NCIN) (100 nM, respectively). Forty-eight hours, the miRNA level was detected by quantitative RT-PCR (A), and Seventy-two hours later, the transfected cells were photographed by a microscope with a CCD camera (bar 100 µm), and the cell numbers were counted using an auto cell counter (B) .

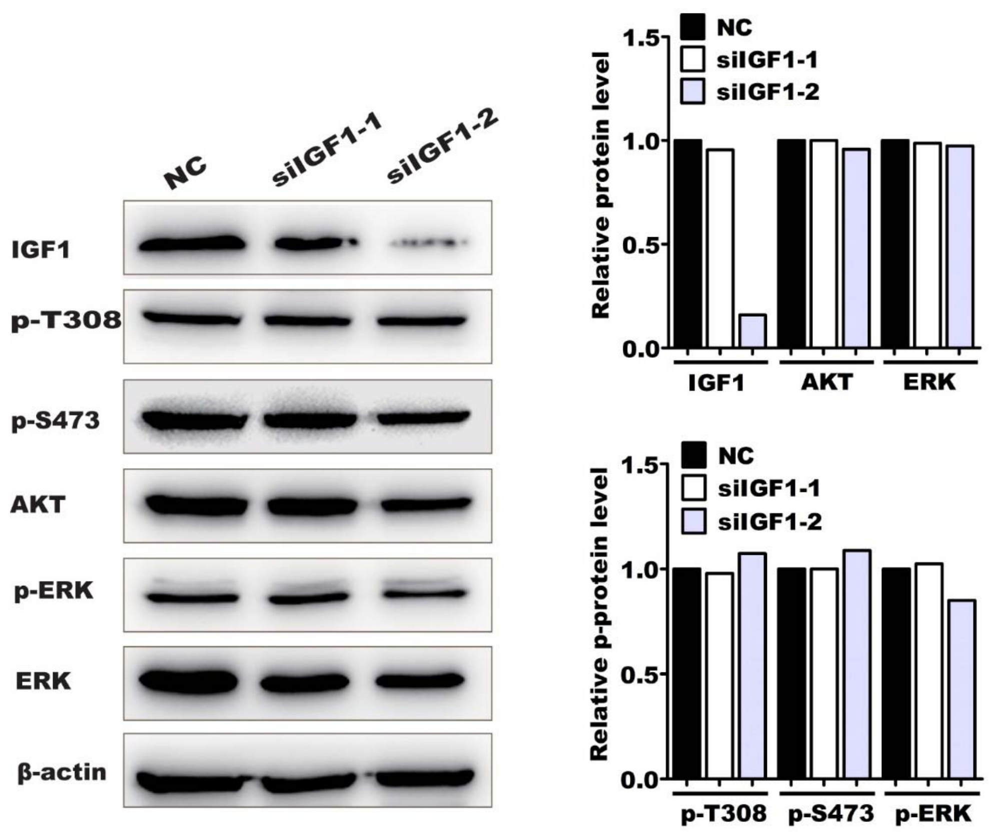

Supplementary Fig. S3. Effect of knocking-down IGF1 on the phosphorylation of AKT and ERK1/2. Pancreatic progenitor cells were transfected with the control siRNA (NC) or siIGF1-1/2. Seventy-two hours after transfection, the total and phosphorylated protein level of AKT (p-T308 and p-S473) and ERK1/2 (p-ERK1/2 at Thr202/Tyr204) were determined by western blot. β-actin was used as a loading control. Uncropped images for the blots were shown in Supplementary Figure S8.

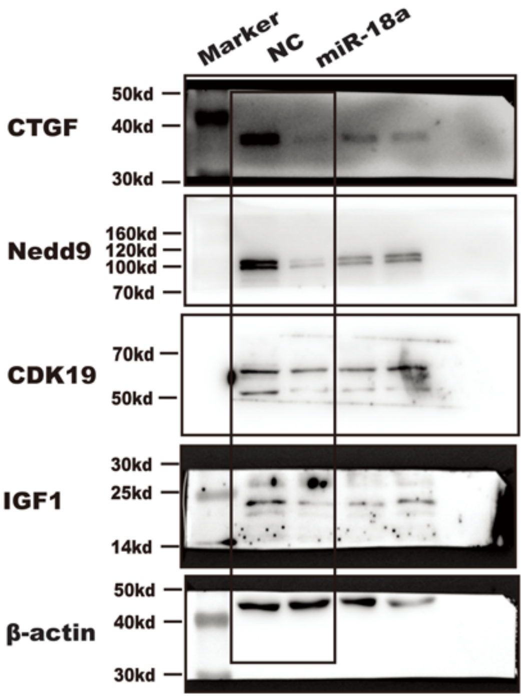

Supplementary Fig. S4. Full-length blot results of Figure 3C.

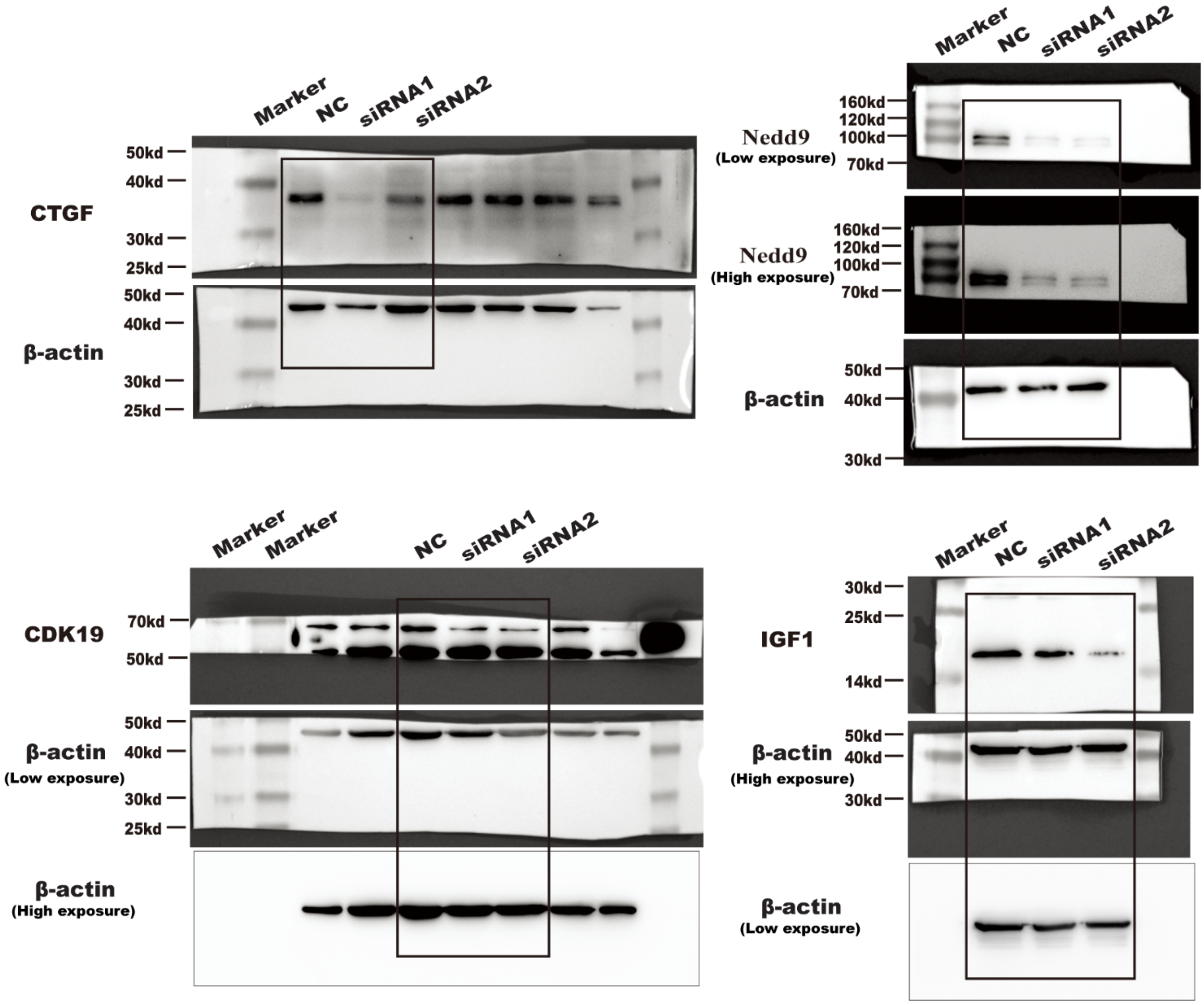

Supplementary Fig. S5. Full-length blot results of Figure 4A

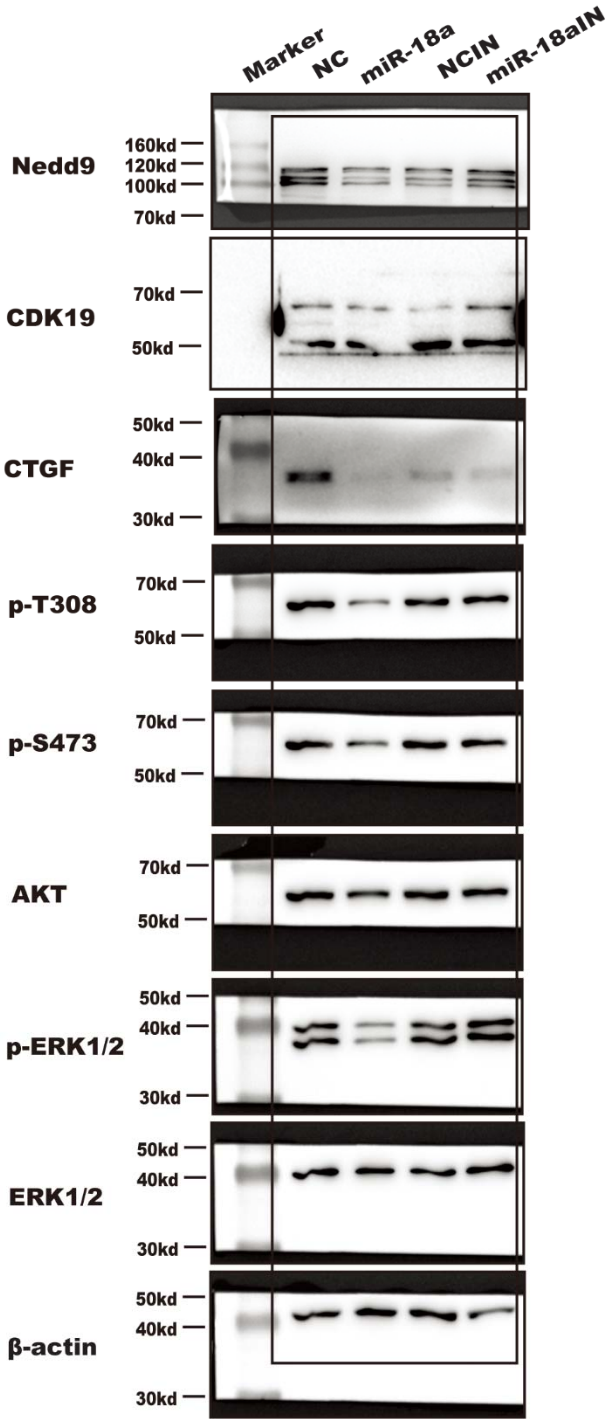

Supplementary Fig. S6. Full-length blot results of Figure 5A.

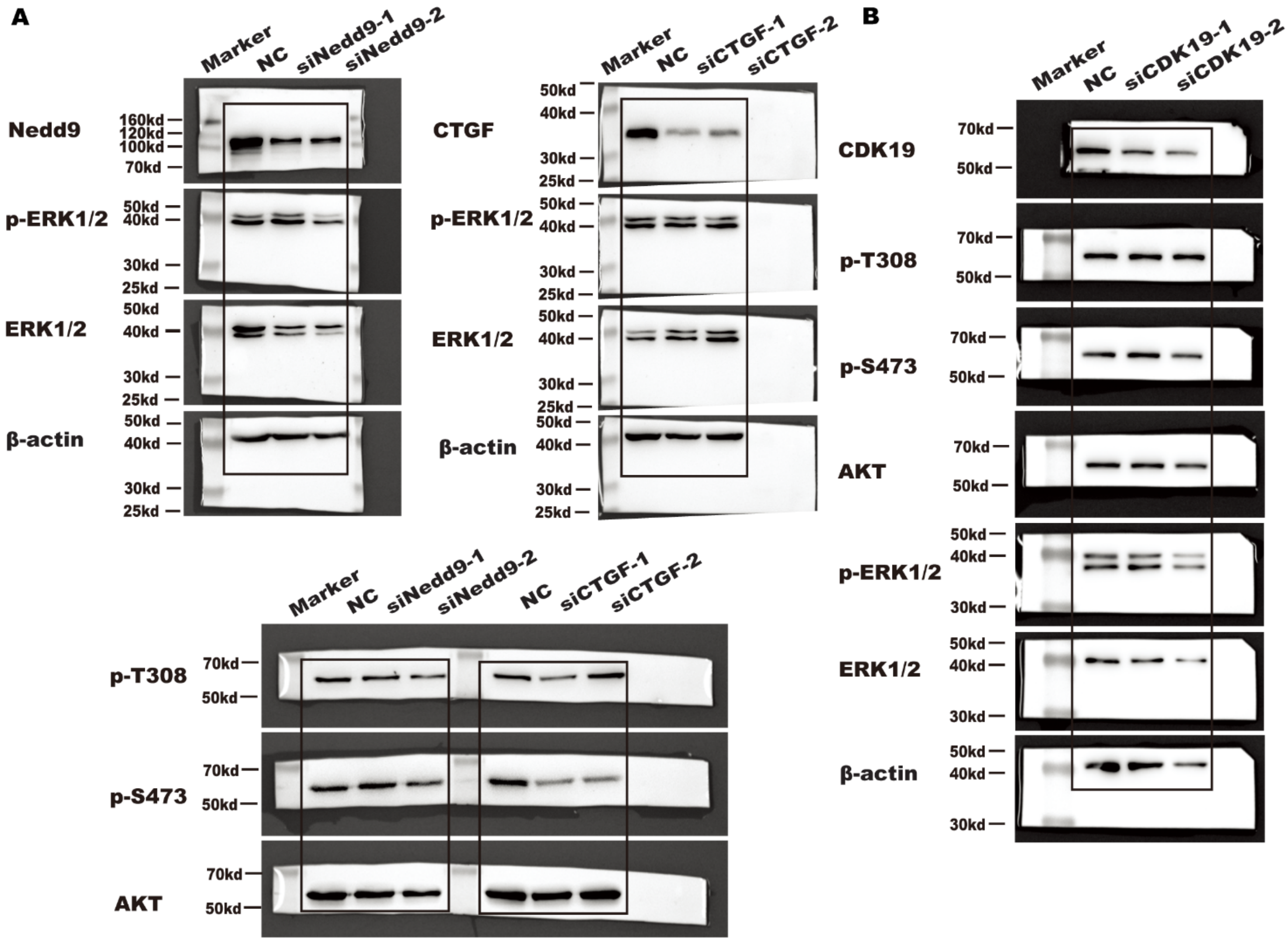

Supplementary Fig. S7. Full-length blots. (A) Blot results of Figure 6A and 6B. (B) Blot results of Figure 6C.

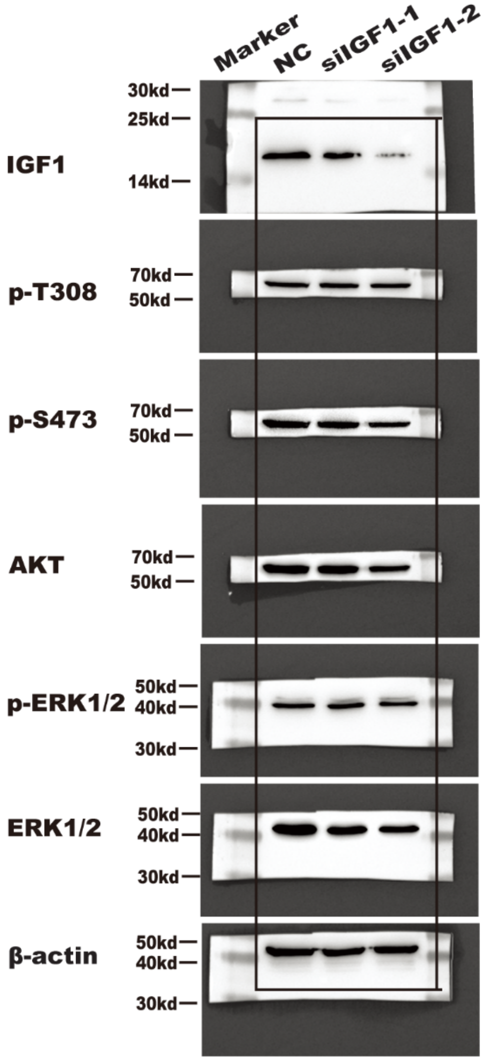

Supplementary Fig. S8. Full-length blot results of Supplementary Figure S3.
